# Laser Cladding of Ultra-Thin Nickel-Based Superalloy Sheets

**DOI:** 10.3390/ma10030279

**Published:** 2017-03-10

**Authors:** Tobias Gabriel, Daniel Rommel, Florian Scherm, Marek Gorywoda, Uwe Glatzel

**Affiliations:** 1 Metals and Alloys, University Bayreuth, Ludwig-Thoma-Straße 36b, 95447 Bayreuth, Germany; tobias.gabriel@uni-bayreuth.de (T.G.); daniel.rommel@uni-bayreuth.de (D.R.); florian.scherm@uni-bayreuth.de (F.S.); 2 Materials Engineering, University of Applied Sciences Hof, Alfons-Goppel-Platz 1, 95028 Hof, Germany; marek.gorywoda@hof-university.de

**Keywords:** laser cladding, selective coating, microstructural characterization, thin sheet material

## Abstract

Laser cladding is a well-established process to apply coatings on metals. However, on substrates considerably thinner than 1 mm it is only rarely described in the literature. In this work 200 µm thin sheets of nickel-based superalloy 718 are coated with a powder of a cobalt-based alloy, Co–28Cr–9W–1.5Si, by laser cladding. The process window is very narrow, therefore, a precisely controlled Yb fiber laser was used. To minimize the input of energy into the substrate, lines were deposited by setting single overlapping points. In a design of experiments (DoE) study, the process parameters of laser power, laser spot area, step size, exposure time, and solidification time were varied and optimized by examining the clad width, weld penetration, and alloying depth. The microstructure of the samples was investigated by optical microscope (OM) and scanning electron microscopy (SEM), combined with electron backscatter diffraction (EBSD) and energy dispersive X-ray spectroscopy (EDX). Similarly to laser cladding of thicker substrates, the laser power shows the highest influence on the resulting clad. With a higher laser power, the clad width and alloying depth increase, and with a larger laser spot area the weld penetration decreases. If the process parameters are controlled precisely, laser cladding of such thin sheets is manageable.

## 1. Introduction

In industrial applications, coatings are commonly used to protect metallic components from different types of disadvantageous mechanisms. Depending on the circumstances, the components are coated either completely or only selectively in areas of high demand. For both methods, laser cladding offers the following advantages: the generated coatings are metallurgically bonded to the substrate, the precision and the geometrical freedom are high, and the heat input is localized only to the areas where the component is coated. Therefore, the heat-affected zone (HAZ) is small [1] and, in this work, not even detectable.

There are two different techniques to produce a coating by laser cladding: in the one-step method, the coating material is brought directly into the laser beam. Typically, metallic powders, pastes, or wires are used. In the two-step method, the coating material is pre deposited on the surface of the substrate and, thereafter, exposed to the laser beam. In this case, mostly powders are used [1].

The technology is well established in cladding of turbine blades. The literature deals with different aspects of laser cladding; in some cases the clad is used as a repair technology [2] while, in others, it protects the blades against oxidation [3] or wear [4].

In addition to the laser process parameters, the geometry of the substrate strongly influences the achievable quality of the coating. Published studies typically focus on the investigation of coatings produced on rather massive cylinders [5,6] or plates [7,8,9] as substrates. To our knowledge, only relatively few studies have been carried out on substrates thinner than 10.0 mm [10,11]. 

One of the few studies conducted on substrates thinner than 1.0 mm is described by Burmester et al. [12]: they coated 0.1–0.15 mm thin steel sheets with two different powders of NiCrSiB alloys. Due to the low thickness of the substrates, their Nd:YAG laser was not suitable to generate the coating in continuous wave (CW) operation mode. To minimize the energy input, the Nd:YAG laser was operated in pulsed mode and the substrates had to be cooled by a mini chiller. The resulting microstructure has not been described in detail. However, it has been mentioned that, different to laser cladding on thicker substrates, there are only two, instead of five, different zones found in the cross-section of the clads: an upper zone with many small unmelted particles distributed in the clad, and the main microstructure with rather coarse grains and, to some extent, equiaxed crystals [12]. A major concern is the fact that, in contrast to thick substrates, the heat dissipation is only two dimensional. The energy brought in by the laser can only conduct in the planar direction within the sheet. This leads to a pronounced distortion of the processed and unprocessed sheet. The distortion of the substrate also affects the stability of the melt pool as it changes the effective size of the laser beam diameter irregularly [12].

The objective of the present work is the fabrication and investigation of coatings on 0.2 mm thin substrates by laser cladding. The optimum set of process parameters is established by using a design of experiments (DoE) procedure. The microstructure of the system (substrate and clad) is observed. The chosen process method is the one-step cladding with a laterally-positioned nozzle. Active cooling of the substrates is not used in order that the results can easily be transferred to different, more complex, geometries.

## 2. Materials and Methods

### 2.1. Materials

**Figure 1:**
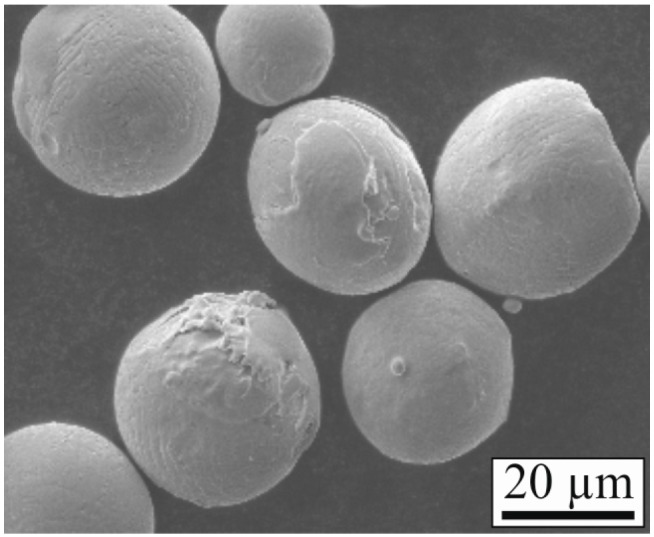
SEM picture of the powder used as coating material.

Flat sheet material of the nickel-based superalloy 718 was selected as the substrate. As the coating material, a powder of a cobalt-based alloy was chosen. The composition of both materials is listed in Table 1. Their melting ranges are similar to each other with 1260–1336 °C (Alloy 718) and 1320–1420 °C (Co-based alloy). Cobalt- and nickel-based alloys are commonly used materials for high-temperature applications, in which coatings are often used to protect components from wear. Cobalt-based alloys show good wear resistance and weldability. Both materials are well described in the literature and there is extensive experimental data in their laser processing. Previous work with these materials at the University Bayreuth, Metals and Alloys include Rommel et al. [13], Strößner et al. [14], and Trosch et al. [15], for example. The specimens for the laser cladding process are of dimensions 40 mm × 20 mm × 0.2 mm, the initial particle size of the gas-atomized powder (Dentaurum, Ispringen, Germany) is 10–30 µm in diameter. For safety reasons, the powder is sieved and only the fraction of 20–30 µm is used. Figure 1 shows a SEM picture of the coating material powder.

### 2.2. Laser Cladding Setup and Process

The laser cladding setup consisted of four main components: laser source, powder delivery, positioning system and housing.

**Figure 2:**
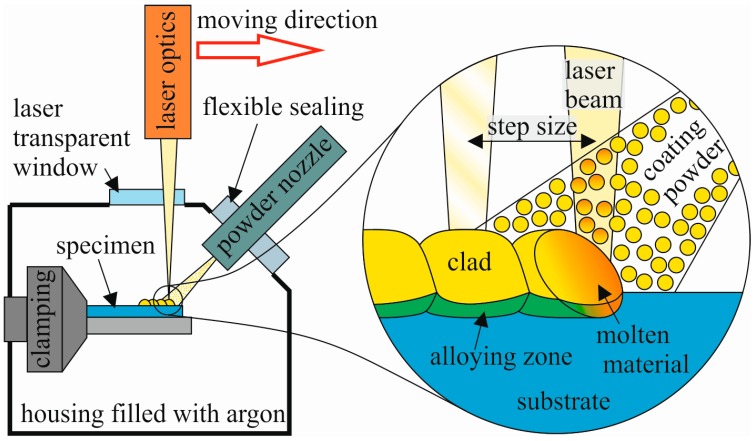
Schematic laser cladding setup.

As the laser source, an Yb fiber laser (YLR-150/1500-QCW-MM by IPG Photonics, Burbach, Germany) with a wavelength of 1070 nm was used. It offered a minimum output of 20 W and a maximum output of 250 W in CW mode. A pneumatic powder feeder (TWIN 10 C by Sulzer Metco, Winterthur, Switzerland) was used for powder delivery. Argon 5.0 transported the powder to the powder nozzle with a flow rate of 4 L/min. The powder feed rate was 8 g/min. Both the laser optics and the powder nozzle were mounted to a three-axis linear positioning system (Minirot 3.1 by Steigerwald Strahltechnik, Maisach, Germany). The powder nozzle was designed and built at the workshop of the University Bayreuth, Metals and Alloys. It was installed in an angle of 45° and with a distance of 10 mm to the substrate surface. Cladding was fabricated with trailing powder nozzle by setting single overlapping points. In order to avoid oxidation of the substrate and the coating, a self-constructed housing equipped with an oxygen sensor was used. It was flooded with argon 5.0. The laser cladding process was started as soon as the oxygen concentration in the housing dropped below 100 ppm. A schematic sketch of the laser cladding setup can be seen in Figure 2.

To generate the line coating on the substrate, the laser beam was turned on for several tenths of a second (exposure time), so that it melted the substrate only to a preferably low depth and most of the powder particles. In this manner, a small round point was produced after solidification of the molten material. The positioning system then moved one step further in order to set the next point with a small overlap on the previous one. For one coated line of approximately 7 mm in length, 14 or 18 points, depending on the step size, are required.

Process parameters were optimized by following a two-level fractional factorial DoE procedure [16], designed with Minitab 17 (Version 17.2.1, Minitab, State College, PA, USA). The process parameters that were varied were laser power, laser spot area (via a variation of the working distance), and traverse speed. More precisely, the traverse speed is not a directly-adjustable parameter, but it depends on the step size, the exposure time for one single point, and the solidification time. With changing the working distance, the laser spot area and, thus, the energy density, is adjusted. Fixed parameters were the powder feed rate, shielding gas flow rate, and distance and angle between the powder nozzle and the substrate surface. Table 2 lists the process parameters and derivative dimensions used in this work.

With five varied process parameters and a design resolution of V, 16 different combinations of parameter settings had to be investigated. With each parameter set, four specimens were produced so that the total number of specimens was equal to 64. The notation of the specimens was carried out according to the following procedure: the 16 parameter sets were named C1 to C16, and the four specimens of each set were labeled from −1 to −4.

### 2.3. Characterization Methods and Specimen Preparation

**Figure 3:**
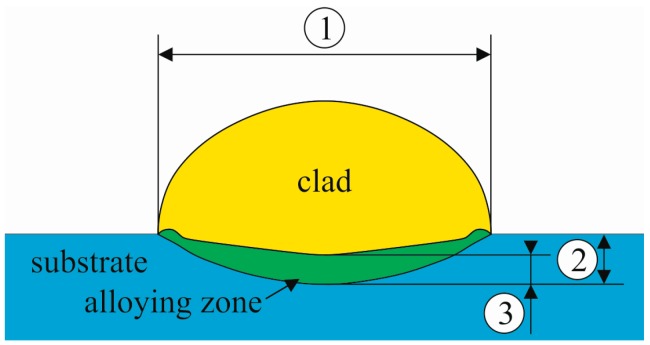
Definition of the quality criteria clad width ①, weld penetration ②, and alloying depth ③.

The quality of the claddings was examined according to four criteria: the clad width, the weld penetration, the alloying depth, and the microstructure of the coating, the substrate, and their interface. Except the microstructure, which was assessed qualitatively, all other criteria can be measured quantitatively. These quantities are schematically shown in Figure 3. For each parameter set, the mean of four specimens was calculated to determine the value of the particular quality criterion.

**Figure 4:**
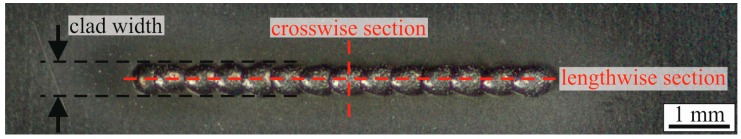
Position of the cross-sections and the measurement of the clad width.

The clad width was measured with an optical microscope (OM, Axioplan 2 by Zeiss, Oberkochen, Germany) on “as clad” samples after cleaning the surface with ethanol and without further preparation (see Figure 4). A large width of the clad line was assessed as positive.

The other criteria, the weld penetration, the alloying depth, and the microstructure, were investigated by scanning electron microscopy (SEM, 1540EsB Cross Beam with FIB and EDX by Zeiss, Oberkochen, Germany), electron backscatter diffraction (EBSD, Sigma 300 by Zeiss, Oberkochen, Germany), and energy dispersive X-ray spectroscopy (EDX, UltraDry by Thermo Fisher Scientific, Waltham, MA, USA). The specimens were prepared by cutting them crosswise and lengthwise to the coating direction. After that, they were embedded in epoxy, ground manually, and polished automatically. Due to the small thickness of the specimens, a smaller weld penetration was assessed as positive. In general, a low alloying of the coating and substrate elements is regarded as being positive [1]. However, as the coating material has to have a certain extent of alloying with the substrate in order to show good bonding properties, and all alloying depths in this work are only in the range of 20–70 µm, the larger alloying depth was chosen to be positive. The microstructures of the coating, substrate, and their interface was examined qualitatively. Figure 4 shows the positions of the cross-sections and of the measurement of the clad width.

## 3. Results and Discussion

From 16 parameter sets, eight led to very good and uniform coatings. Three sets showed good, but inconsistent, results over all four specimens investigated in the set. The coated lines in one, respectively, two specimens were only weakly bonded to the substrate. The other five sets either did not show good coatings or were too irregular. All specimens showed only little distortion after removing them from the cladding setup and no visible cracks were detected.

### 3.1. Quantitative and Statistical Results

**Figure 5:**
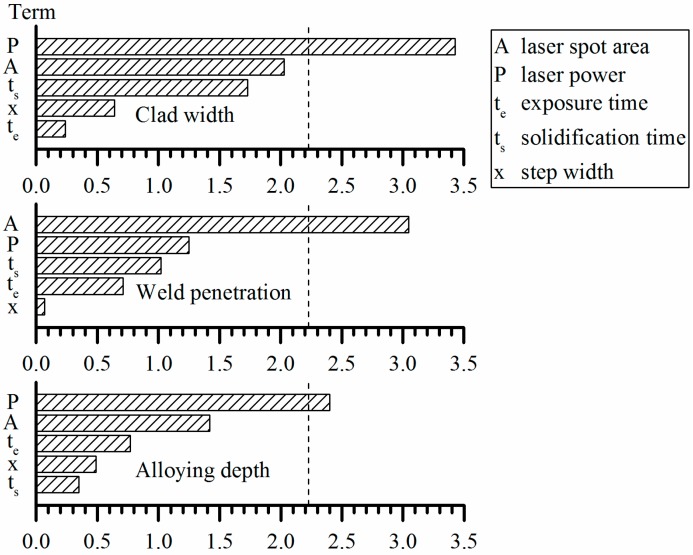
Pareto charts of the standardized effects of the five varied parameters. Statistical significance is given for values higher than the vertical, dashed line.

For the statistical evaluation of the quantitative results (clad width, weld penetration, and alloying depth), all 64 specimens of the 16 parameter sets were taken into account. The analysis showed a quite large scatter of the results. Apparently, the pointwise cladding procedure induces a significant variability of the clad quality. The evaluation was, thus, restricted to the main factors of the process. To find out which process parameters significantly affected the particular result, three Pareto charts of the standardized effects were created. These charts focus on the representation of single parameters without consideration of the interactions between them. The small *p*-values of the laser power and laser spot area in Table 3, and the Pareto charts in Figure 5, show that the statistically significant terms are:

- with respect to the clad width: the laser power
- with respect to the weld penetration: the laser spot area
- with respect to the alloying depth: the laser power

Thus, there are only two process parameters which are significant: the laser power and the laser spot area. Their influence on the quantitative quality criteria can be seen in Table 4. The large standard deviation indicates that more replications of each parameter set would improve the determination of the influence of the single process parameters.

With a higher laser power, the clad width increases. More of the coating and substrate material is molten and solidifies to a droplet. This correlation was expected, as it is the same behavior as described by Del Val et al. [9] for laser cladding on thicker substrates. In this work, the width of well-fabricated coated lines was always larger than the thickness of the substrate with a minimum of 342 µm and a maximum of 620 µm.

With a higher laser spot area, the weld penetration decreases. The same amount of energy is brought into the substrate with decreasing energy density and less substrate material is molten. Burmester et al. [12] state that the thickness of the substrate has to be at least 3–5 times that of the weld penetration in order to obtain good cooling characteristics. In rather good accordance to that, we achieved good coatings with a ratio of minimum 2.5 of substrate thickness to weld penetration.

With a higher laser power, the alloying depth increases. The higher energy input into the substrate leads to higher temperatures and, therefore, the melt pool dynamic increases [17]. If the coated line is welded properly with the substrate, the alloying depth lies between 1 µm and 70 µm.

**Figure 6:**
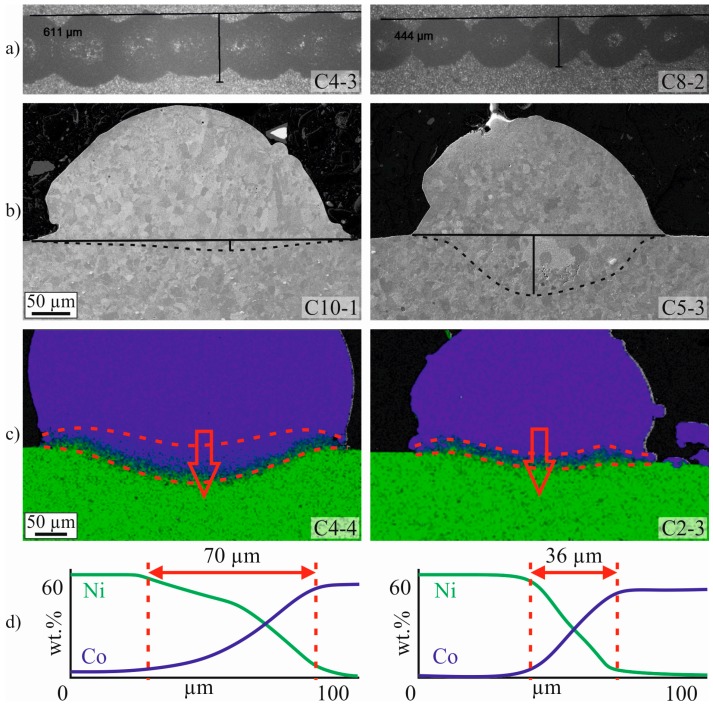
(**a**) Clad width (OM); (**b**) weld penetration (SEM Secondary Electron (SE)); (**c**) alloying depth (EDX image); and (**d**) EDX line scan. Co is shown in blue, Ni in green. On the left, an example of a good weld is shown and, on the right, a poor one.

Figure 6a shows one example of a large clad width on the left (sample C4-3) and a small one on the right (C8-2). In Figure 6b one example of a small weld penetration on the left (C10-1) and a large one on the right (C5-3) is shown. Figure 6c shows one example of a large alloying depth on the left (C4-4) and a small one on the right (C2-3), the corresponding EDX line scans across the weld interaction is shown in Figure 6d.

The statistical analysis was subsequently used to conduct a response optimization. This leads to the prediction of the best parameter set with respect to the resulting clad width, weld penetration, and alloying depth. The closest match to these parameters were used with sets C4 and C12, whereas it was found that the set C12 is less stable than C4. Table 5 compares the optimum predicted parameters and predicted values of the clad width, the weld penetration, and the alloying depth with the parameters and results of the experimentally-investigated parameter set C4. A close agreement between the prediction and the experiment is found.

### 3.2. Microstructural Analysis

**Figure 7:**
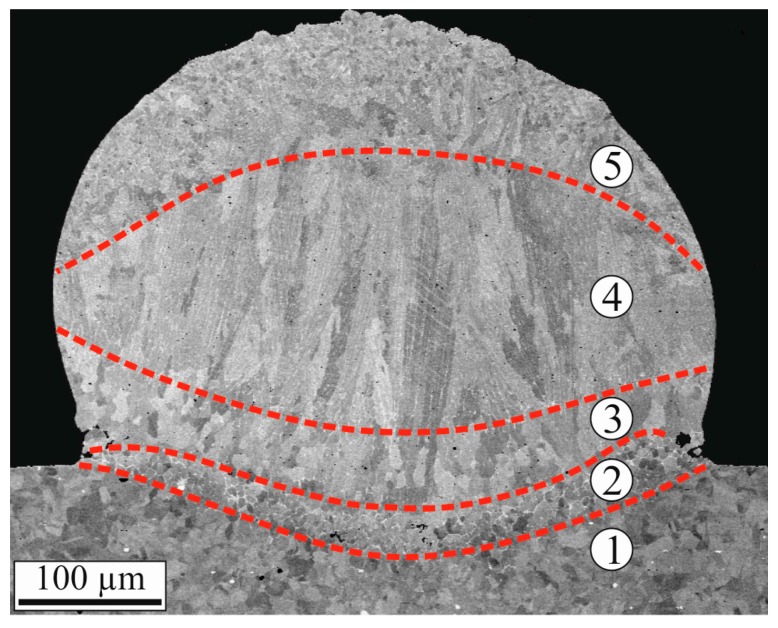
Cross-section of the specimen C4-4 (SEM BSE). Five different areas, numbered from 1 to 5, can be recognized in the microstructure of the clad.

Most SEM images were taken on cross-sections. In principle, the microstructure looks the same in all specimens for a well-fabricated clad. Figure 7 shows a SEM Backscattered Electron (BSE) image of specimen C4-4, in which five different areas were marked. The areas numbered 2–4 are in good accordance with the four zones defined by Zhong and Liu [18] for thicker substrates, and are in contrast to Burmester et al. [12], where only two zones were found in 0.1 to 0.15 mm thin substrates.

The microstructure of the substrate did not change in area 1 and no distinct heat-affected zone can be seen. This is in contrast to thicker substrates and higher laser power, in which heat affected zones are observed [7,10]. In the molten, and then solidified, area 2, the grains are slightly smaller than in the substrate, with an average grain diameter of about 7 µm. The substrate and coating materials have intermixed in area 2 and alloyed with each other. The composition is closer to that of the substrate than that of the coating. At the grain boundaries, an enrichment of Nb is found. In addition, some porosity can be seen in this zone.

Area 3 also belongs to the alloying zone. However, in area 3 the composition is closer to that of the coating material. The grains are elongated in the perpendicular direction to the boundary of the melt pool and larger than in area 2; they are about 15–20 µm long.

In area 4, most of the grains are approximately 30 to 100 µm long and highly orientated, again, perpendicularly to the boundary of the melt pool.

Finally, area 5 contains very fine grains with an average grain diameter of about 4 µm. In some cases, these fine grains are elongated perpendicularly and radially to the molten powder particles of the coating material. Occasionally, unmelted powder particles could be found in the outer edge of the melt pool and, in a few cases, the powder particles were distributed all over the clad area. The lengthwise sections show the same areas, but there, the contact to the surface of the substrate is smaller because every cladded point is overlapped by a successive one.

**Figure 8:**
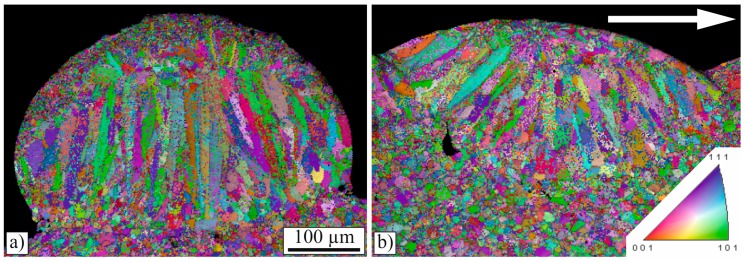
(**a**) Cross-section and (**b**) lengthwise section combined image quality and orientation map (EBSD). On the lengthwise section, cladding direction is from left to right.

In contrast to conventional continuous laser cladding, the clad in this work has been produced in a static manner, as the positioning system did not move while the laser beam was turned on. Therefore, the microstructure was not expected to have different cross- and lengthwise orientations with respect to the cladding direction. To verify this, EBSD image quality maps were generated from both the cross- and the lengthwise section, as shown in Figure 8. It can be seen that the orientation of the elongated grains is, in both cases, perpendicular to the boundary of the melt pool and that the grains do not have any crystallographic preferred orientation. The elongation of the grains in the direction perpendicular to the melt pool agrees with the findings of Ocelík et al. [5] and Farnia et al. [10]. In contrast to these publications, in this work, the highly-longitudinal orientated grains occupied more than 70% of the clad height in the crosswise sections instead of approximately 20% [5], or 50% [10]. This difference is attributed to two facts: due to the low heat dissipation into the thin substrate, the grains tend to grow in the direction perpendicular to the surface, and in addition to this, a conventional continuously-pulsed cladding method was used in these studies and the elongated grains were, thus, inclined to the cladding direction [5,10]. In lengthwise cross-sections, the elongated grains take up 80% of the clad height [5,10]. The orientation perpendicular to the melt pool results from the growing direction of the grains because the heat transfer during solidification is perpendicular to the solidification front [5].

**Figure 9:**
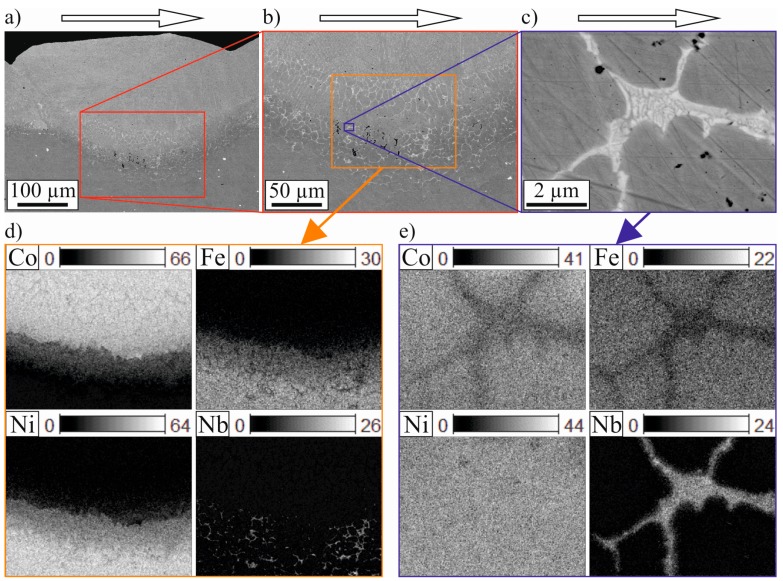
Lengthwise section of a coated line (SEM BSE) with increasing magnification from (**a**) to (**c**), cladding direction is from left to right; (**d**) elemental distribution of the transition between substrate and coating material; and (**e**) detail of elemental distribution with Nb-enriched zones in area 2 (both EDX, wt%). These Nb-enriched zones are identified as Laves phase.

The nickel-based superalloy 718 and its properties are the subject of many studies, however, it is rarely used as a substrate material in laser cladding. More often it is subjected to selective laser melting. The microstructure of in such a way processed alloy 718 is well described [14,15]. Figure 9 shows EDX images of a lengthwise section and details of area 2. Strößner et al. [14] describe, that in a laser molten alloy 718, Nb enriches inter- and intragranularly and forms a plate-like δ-phase (Ni_3_Nb). In this work, the process differs, but as can be seen in Figure 9, an intergranular Nb enrichment is identified, although not plate-like. The Nb-rich zones contain approximately 12 at% Ni, 9 at% Fe, 32 at% Cr, 21 at% Nb, and 11 at% Ti. This gives a ratio of (Ni, Fe, Cr):(Nb, Ti) of 1.7:1, which is in good agreement with the theoretical ratio of a Laves phase ((Ni, Fe, Cr)_2_ (Nb, Mo, Ti)) [19]. The appearance in this work is similar to the Laves phase described in welded Inconel 718 by Vincent [20]. He detected a comparable composition to the one in this work, if one takes the absence of the second alloy into account. His Ni content in the Laves phase is approximately as high as the combined Co and Ni concentration found in this work. The formation of the Laves phase was described by Radhakrishna et al. [19,21]. The Laves phase forms due to microsegregation of the refractory element Nb, which tends to segregate during solidification. A low heat input and high heat dissipation lead to a reduced Laves phase formation [21]. Despite the fact that the heat input in this work was relatively low and localized, the heat dissipation was poor due to the small thickness of the substrate and the uncooled laser cladding setup, which facilitated the formation of a Laves phase.

**Figure 10:**
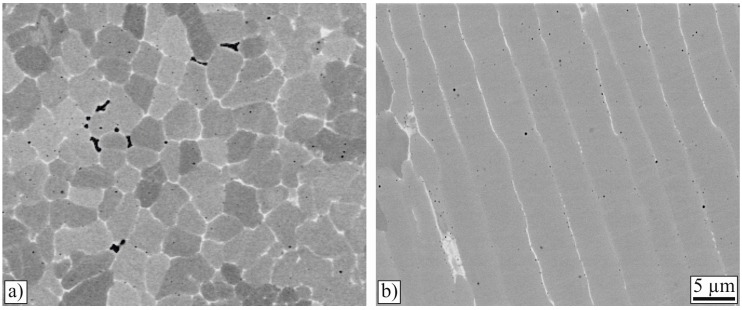
(**a**) Intergranular enrichments of Si, Cr, and W (light gray) in area 5; and (**b**) line-orientated inter- and intragranular enrichments in area 4 (both SEM BSE).

In the areas 4 and 5, Si-, Cr-, and W-enriched zones were detected. In these zones, the Co concentration is reduced (from nominal 61.2 at% to approximately 51.9 at%), while the Si, Cr, and W concentrations are slightly increased (from nominal 3.2, 32.1, and 2.9 at% to approximately 5.6, 34.5, and 5.1 at%). According to the literature, Cr_3_Si can be found as phase in wear resistant alloys. Under high solidification rates, as encountered in laser cladding, Cr_3_Si is known to precipitate from the liquid phase and to grow in parallel straight lines, as described by Wang and Duan [22]. In area 4, this orientation can be seen, as shown in Figure 10. In area 5, the composition analyses indicate that the bright zones at the grain boundaries are enriched in Si and Cr and, thus, contain Cr_3_Si.

## 4. Conclusions

Very thin sheets of only 200 µm of a nickel-based superalloy can be coated by laser cladding with a Co-based alloy. To minimize the input of energy into the substrate, the coated lines are generated by a pointwise cladding. The results can be summarized as follows:

1. The 200 µm thin substrates were coated successfully. Optima for the laser power, the laser spot area, the step size, the exposure time, and the solidification time were found.
2. The microstructure of well-fabricated clads looks very similar over all of the process parameters. It is comparable to that of clads of thick substrates described in the literature. Five different areas can be identified:
  - Substrate without changes and no distinct heat affected zone.
  - Molten and solidified material with a composition close to the substrate material. The grains are smaller and the grain boundaries are enriched with Nb. This Nb enrichment is identified as a Laves phase.
  - Slightly elongated grains, orientated perpendicularly to the solidification front.
  - Highly elongated grains, orientated perpendicularly to the solidification front. Inter- and intragranular enrichment of Si and Cr, supposed to be Cr_3_Si.
  - Very fine grains with enrichment of Si and Cr at the grain boundaries, also supposed to be Cr_3_Si.
3. In general, the influence of the process parameters is comparable to laser cladding of thicker substrates. The main effects are:
  - the clad width increases with increasing laser power
  - the weld penetration increases with decreasing laser spot area
  - the alloying depth increases with increasing laser power
4. The process is very sensitive to the process parameters. It turned out that the minimum laser power used in this work is close to the lower limit to generate well-cladded coatings at all. Therefore, another test series with higher laser powers will be planned for future work. More replications of every parameter set will be considered in this test series for more meaningful statistical analysis.

## Figures and Tables

**Table 1:** Composition of the substrate and coating material.

Elements in wt. %	Ni	Cr	C	Mo	Nb	Ti	Fe	Co	W	Si
Substrate	50–55	17–21	0.08 (max.)	2.8–3.3	4.75–5.5	0.65–1.15	14–24 (bal.)	-	-	-
Coating	-	28	-	-	<1	-	-	60.5	9	1.5

**Table 2:** Laser cladding process parameters and derivative dimensions.

Process Parameter	Value		Adjustable	Note
	Min.	Max.		
Laser power (W)	64	82	x	Varied
Working distance (mm)	10	14	x	Varied
Laser spot diameter (mm)	0.7	0.9		Derivative
Laser spot area (mm^2^)	0.38	0.64		Derivative
Energy density (J/mm^2^)	10.00	43.16		Derivative
Step size (mm)	0.4	0.5	x	Varied
Exposure time (s)	0.1	0.2	x	Varied
Solidification time (s)	0.2	0.8	x	Varied
Traverse speed (mm/s)	0.4	1.67		Derivative
Powder feed rate (g/min)	8		x	Fix
Shielding gas flow rate (L/min)	4		x	Fix
Distance nozzle to surface (mm)	10		x	Fix
Angle between nozzle and surface (°)	45		x	Fix
Powder nozzle diameter (mm)	0.85			Fix
Laser beam parameter product (mm·mrad)	0.35			Fix

**Table 3:** Analysis of variance (ANOVA) of the single parameters without interactions. For reasons of clarity, only the *F*- and *p*-values are given. t_e_ is the exposure time, x is the step size, t_s_ is the solidification time, A is the laser spot area, and P is the laser power.

Source	Clad Width		Weld Penetration		Alloying Depth	
	*F*-Value	*p*-Value	*F*-Value	*p*-Value	*F*-Value	*p*-Value
t_e_	0.06	0.813	0.51	0.492	0.59	0.459
x	0.41	0.535	0.01	0.943	0.24	0.635
t_s_	2.99	0.115	1.05	0.329	0.12	0.733
A	4.13	0.069	9.34	0.012	2.02	0.185
P	11.75	0.006	1.57	0.239	5.77	0.037

**Table 4:** Influence of laser power on mean clad width, mean alloying depth, and of the spot area on mean weld penetration. The values in brackets give the standard deviation.

Laser Power (W)	Clad Width (µm)	Alloying Depth (µm)	Laser Spot Area (mm^2^)	Weld Penetration (µm)
64	330 (193)	28 (17)	0.38	39 (13)
82	558 (38)	47 (9)	0.64	17 (13)

**Table 5:** Parameters and (predicted) results of optimized parameter set and set C4.

		Predicted Optimum	C4
Parameters	Laser power (W)	82	82
	Laser spot area (mm^2^)	0.64	0.38
	Step size (mm)	0.5	0.5
	Exposure time (s)	0.2	0.2
	Solidification time (s)	0.2	0.5
Results	Clad width (µm)	577	602
	Weld penetration (µm)	23	57
	Alloying depth (µm)	47	60
